# (Benzonitrile-κ*N*)chlorido[hydrido­tris(pyrazol-1-yl-κ*N* ^2^)borato](triphenyl­phosphine-κ*P*)ruthenium(II) ethanol solvate

**DOI:** 10.1107/S1600536809010265

**Published:** 2009-03-25

**Authors:** Hung-Chun Tong, Yu-Chen Hung, Po-Yo Wang, Chia-Her Lin, Yih-Hsing Lo

**Affiliations:** a Department of Chemical Engineering, Tatung University, Taipei 104, Taiwan; b Department of Natural Science, Taipei Municipal University of Education, Taipei 10048, Taiwan; c Department of Chemistry, Chung-Yuan Christian University, Chung-Li 320, Taiwan

## Abstract

The reaction of [Ru(C_9_H_10_BN_6_)Cl(C_18_H_15_P)_2_] with benzo­nitrile leads to crystals of the title compound, [Ru(C_9_H_10_BN_6_)Cl(C_18_H_15_P)(C_7_H_5_N)]·C_2_H_5_OH. In the crystal structure, the environment about the ruthenium metal center corresponds to a slightly distorted octa­hedron with an average N—Ru—N bite angle of the Tp ligand of 86.6 (2)°.

## Related literature

For general background to the hydridotris(pyrazoly)borate anion and its use in the preparation of various transition metal complexes, see: Alcock *et al.* (1992 ▶); Burrows *et al.* (2001 ▶); Pavlik *et al.* (2005 ▶); Slugovc *et al.* (1998 ▶); Trofimenko (1993 ▶). For Ru—N distances in other hydridotripyrazolyl­borate complexes, see: Gemel *et al.* (1996 ▶); Slugovc *et al.* (1998 ▶).
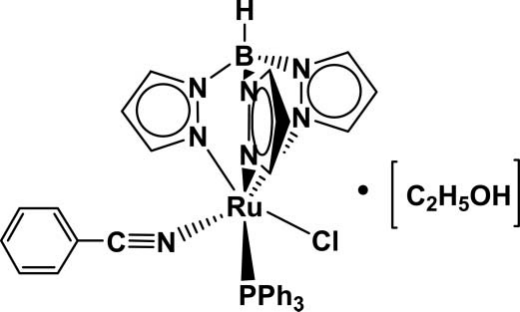


## Experimental

### 

#### Crystal data

[Ru(C_9_H_10_BN_6_)Cl(C_18_H_15_P)(C_7_H_5_N)]·C_2_H_6_O
*M* *_r_* = 761.02
Triclinic, 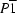

*a* = 8.0008 (5) Å
*b* = 11.0195 (5) Å
*c* = 19.4246 (11) Å
α = 83.438 (4)°
β = 88.726 (4)°
γ = 88.920 (4)°
*V* = 1700.70 (16) Å^3^
*Z* = 2
Mo *K*α radiation
μ = 0.63 mm^−1^
*T* = 200 K
0.24 × 0.08 × 0.02 mm

#### Data collection

Nonius KappaCCD diffractometer
Absorption correction: multi-scan (Blessing, 1995 ▶) *T*_min_ = 0.864, *T*_max_ = 0.988
13842 measured reflections
5819 independent reflections
3804 reflections with *I* > 2σ(*I*)
*R*_int_ = 0.080

#### Refinement

*R*[*F* ^2^ > 2σ(*F* ^2^)] = 0.056
*wR*(*F* ^2^) = 0.122
*S* = 1.04
5819 reflections
433 parameters
H-atom parameters constrained
Δρ_max_ = 0.86 e Å^−3^
Δρ_min_ = −0.89 e Å^−3^

### 

Data collection: *COLLECT* (Nonius, 1999 ▶); cell refinement: *DENZO* and *SCALEPACK* (Otwinowski & Minor, 1997 ▶); data reduction: *DENZO* and *SCALEPACK*; program(s) used to solve structure: *SHELXS97* (Sheldrick, 2008 ▶); program(s) used to refine structure: *SHELXL97* (Sheldrick, 2008 ▶); molecular graphics: *ORTEP-3 for Windows* (Farrugia, 1997 ▶); software used to prepare material for publication: *WinGX* (Farrugia, 1999 ▶).

## Supplementary Material

Crystal structure: contains datablocks I, global. DOI: 10.1107/S1600536809010265/nc2139sup1.cif

Structure factors: contains datablocks I. DOI: 10.1107/S1600536809010265/nc2139Isup2.hkl

Additional supplementary materials:  crystallographic information; 3D view; checkCIF report

## Figures and Tables

**Fig. 1.**
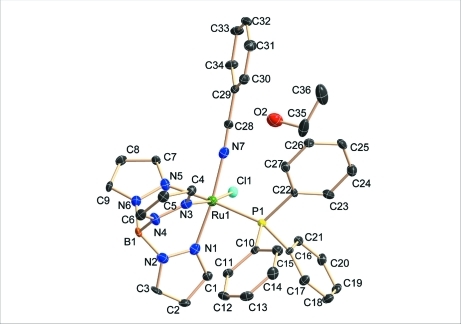
Molecular structure of (the title compound with labelling and displacement ellipsoids drawn at the 30% probability level (H atoms are shown as spheres of arbitrary radius).

## supplementary crystallographic information

### Comment

Hydridotris(pyrazoly)borate anion (Tp,HB(pz)_3_) has been introduced by Trofimenko as a ligand in the preparation of various transition metal complexes (Trofimenko, 1993). Ruthenium(II) hydridotripyrazolylborate complexes, Ru(Tp), are of interest for stoichiometric and catalytic transformations of organic molecules (Pavlik *et al.*, 2005). The complex [Ru(Tp)Cl(PPh_3_)_2_] (Alcock *et al.*, 1992) has been used as the starting material for the synthesis of several complexes because of its substitutionally labile chloride and phosphine substitutents (Burrows, 2001). On the other hand, Benzonitrile can form coordination complex with late transition metals that are both soluble in organic solvents and conveniently labile, *e.g.* PdCl_2_(PhCN)_2_. The benzonitrile ligands are readily displaced by stronger ligands, making benzonitrile complexes useful as synthetic intermediates.

In the crystal structure of the title compound the ruthenium metal center is coordinated by four N, one P and one Cl atom within slightly distorted octahedra. The  average N—Ru—N bite angle to the Tp ligand amount to 86.6 (2)°. The three Ru—N(Tp) bond lengths of 2.063 (5), 2.069 (4) and 2.102 (5) Å) are slightly longer than the average distance of 2.038 Å in other ruthenium Tp complexes (Gemel *et al.* 1996; Slugovc *et al.* 1998). The Ru1—N7 and N7—C28 bond lengths of 1.991 (5) Å and 1.144 (7)Å correspond to single Ru—N and C≡N bonds. The crystal structure conatin additional ethanol molecules, which are connected to the chloro atom via weak O-H···Cl hydrogen bonding.

### Experimental

To a solution of [Ru(Tp)Cl(PPh_3_)_2_](3.95 g,4.50 mmol) in toulene (100 ml), an excess of benzonitrile (4.6 ml, 45.0 mmol) was added. The mixture was heated using a warm water bath for 30 min. A deep yellow color developed during this time. The reaction mixture was stirred for a further 2 h at room temperature (298 K). Then it was concentrated to approximately half of the volume and cooled to 273 K. The yellow precipitate was filtered off, washed with ethanol and ether and dried was dried under vacuum to give the title compound (I) (2.09 g, 65% yield).The bright-yellow crystals of (I) for X-ray structure analysis were obtained by recrystallization of the crude product from dichloromethane–ethanol containing free benzonitrile. The IR spectra display one medium band near 2214 cm^-1^, which was assigned to the ν(CN) vibration of the nitrile ligand. We have observed that the benzonitrile is lost readily in solution. Therefore, in all perations for the purification of the title compound, an excess of free nitrile was added in order to prevent the nitrile ligand dissociation.

### Refinement

The H atoms were placed in idealized positions and constrained to ride on their parent atoms, with C—H = 0.95 - 0.99 Å and *U*_iso_(H) = 1.2 or 1.5*U*_eq_(C), B—H = 1.0 Å and *U*_iso_(H) = 1.2*U*_eq_(B), and O—H = 0.84 Å and *U*_iso_(H) = 1.2*U*_eq_(N).

### Crystal data

**Table:**

[Ru(C_9_H_10_BN_6_)Cl(C_18_H_15_P)(C_7_H_5_N)]·C_2_H_6_O	*Z* = 2
*M**_r_* = 761.02	*F*(000) = 780
Triclinic, *P*1	*D*_x_ = 1.486 Mg m^−^^3^
*a* = 8.0008 (5) Å	Mo *K*α radiation, λ = 0.71073 Å
*b* = 11.0195 (5) Å	Cell parameters from 0 reflections
*c* = 19.4246 (11) Å	θ = 0–0°
α = 83.438 (4)°	µ = 0.63 mm^−^^1^
β = 88.726 (4)°	*T* = 200 K
γ = 88.920 (4)°	Prism, yellow
*V* = 1700.70 (16) Å^3^	0.24 × 0.08 × 0.02 mm

### Data collection

**Table:**

Nonius KappaCCD diffractometer	5819 independent reflections
Radiation source: fine-focus sealed tube	3804 reflections with *I* > 2σ(*I*)
graphite	*R*_int_ = 0.080
Detector resolution: 9 pixels mm^-1^	θ_max_ = 25.0°, θ_min_ = 1.9°
CCD rotation images, thick slices scans	*h* = −7→9
Absorption correction: multi-scan (Blessing, 1995)	*k* = −12→13
*T*_min_ = 0.864, *T*_max_ = 0.988	*l* = −21→23
13842 measured reflections	

### Refinement

**Table:**

Refinement on *F*^2^	Primary atom site location: structure-invariant direct methods
Least-squares matrix: full	Secondary atom site location: difference Fourier map
*R*[*F*^2^ > 2σ(*F*^2^)] = 0.056	Hydrogen site location: inferred from neighbouring sites
*wR*(*F*^2^) = 0.122	H-atom parameters constrained
*S* = 1.04	*w* = 1/[σ^2^(*F*_o_^2^) + (0.0432*P*)^2^] where *P* = (*F*_o_^2^ + 2*F*_c_^2^)/3
5819 reflections	(Δ/σ)_max_ = 0.001
433 parameters	Δρ_max_ = 0.86 e Å^−^^3^
0 restraints	Δρ_min_ = −0.89 e Å^−^^3^

### Special details

**Table:**

Geometry. All e.s.d.'s (except the e.s.d. in the dihedral angle between two l.s. planes) are estimated using the full covariance matrix. The cell e.s.d.'s are taken into account individually in the estimation of e.s.d.'s in distances, angles and torsion angles; correlations between e.s.d.'s in cell parameters are only used when they are defined by crystal symmetry. An approximate (isotropic) treatment of cell e.s.d.'s is used for estimating e.s.d.'s involving l.s. planes.
Refinement. Refinement of *F*^2^ against ALL reflections. The weighted *R*-factor *wR* and goodness of fit *S* are based on *F*^2^, conventional *R*-factors *R* are based on *F*, with *F* set to zero for negative *F*^2^. The threshold expression of *F*^2^ > σ(*F*^2^) is used only for calculating *R*-factors(gt) *etc*. and is not relevant to the choice of reflections for refinement. *R*-factors based on *F*^2^ are statistically about twice as large as those based on *F*, and *R*- factors based on ALL data will be even larger.

### Fractional atomic coordinates and isotropic or equivalent isotropic displacement parameters (Å^2^)

**Table:**

	*x*	*y*	*z*	*U*_iso_*/*U*_eq_	
B1	0.6437 (8)	1.1216 (6)	0.8145 (4)	0.0217 (17)	
H1'	0.6412	1.1988	0.8370	0.026*	
C1	0.4057 (7)	0.8541 (6)	0.8752 (3)	0.0250 (15)	
H1	0.3660	0.7746	0.8709	0.030*	
C2	0.3573 (8)	0.9230 (6)	0.9274 (3)	0.0307 (17)	
H2	0.2812	0.9014	0.9648	0.037*	
C3	0.4430 (8)	1.0291 (6)	0.9132 (3)	0.0292 (17)	
H3	0.4369	1.0963	0.9399	0.035*	
C4	1.0191 (7)	0.9563 (5)	0.7700 (3)	0.0235 (15)	
H4	1.0754	0.8911	0.7507	0.028*	
C5	1.0975 (8)	1.0535 (5)	0.7936 (3)	0.0295 (17)	
H5	1.2141	1.0685	0.7933	0.035*	
C6	0.9697 (8)	1.1240 (6)	0.8178 (3)	0.0298 (17)	
H6	0.9830	1.1977	0.8379	0.036*	
C7	0.4995 (7)	1.0900 (5)	0.6428 (3)	0.0218 (15)	
H7	0.4781	1.0420	0.6064	0.026*	
C8	0.4638 (7)	1.2139 (6)	0.6407 (3)	0.0305 (17)	
H8	0.4164	1.2661	0.6037	0.037*	
C9	0.5113 (7)	1.2444 (5)	0.7030 (3)	0.0232 (15)	
H9	0.5017	1.3234	0.7179	0.028*	
C10	0.8767 (7)	0.7020 (5)	0.8753 (3)	0.0194 (14)	
C11	0.8356 (7)	0.7871 (5)	0.9212 (3)	0.0252 (15)	
H11	0.7450	0.8428	0.9110	0.030*	
C12	0.9232 (8)	0.7922 (6)	0.9807 (3)	0.0300 (17)	
H12	0.8922	0.8506	1.0112	0.036*	
C13	1.0560 (8)	0.7129 (6)	0.9964 (3)	0.0341 (18)	
H13	1.1156	0.7152	1.0380	0.041*	
C14	1.1002 (8)	0.6312 (6)	0.9513 (4)	0.0348 (18)	
H14	1.1937	0.5781	0.9610	0.042*	
C15	1.0116 (7)	0.6240 (6)	0.8916 (3)	0.0299 (17)	
H15	1.0436	0.5651	0.8615	0.036*	
C16	0.5928 (7)	0.5768 (5)	0.8399 (3)	0.0199 (14)	
C17	0.6134 (8)	0.5104 (5)	0.9041 (3)	0.0295 (16)	
H17	0.7035	0.5290	0.9319	0.035*	
C18	0.5063 (8)	0.4179 (6)	0.9288 (4)	0.0333 (17)	
H18	0.5250	0.3731	0.9728	0.040*	
C19	0.3743 (8)	0.3901 (5)	0.8908 (4)	0.0314 (17)	
H19	0.3001	0.3270	0.9081	0.038*	
C20	0.3503 (7)	0.4553 (5)	0.8265 (4)	0.0282 (17)	
H20	0.2592	0.4361	0.7994	0.034*	
C21	0.4563 (7)	0.5477 (5)	0.8013 (3)	0.0247 (15)	
H21	0.4367	0.5921	0.7573	0.030*	
C22	0.8730 (7)	0.5888 (5)	0.7502 (3)	0.0190 (14)	
C23	0.8347 (8)	0.4663 (6)	0.7473 (3)	0.0335 (17)	
H23	0.7423	0.4315	0.7736	0.040*	
C24	0.9301 (8)	0.3950 (6)	0.7066 (4)	0.0367 (18)	
H24	0.9018	0.3121	0.7050	0.044*	
C25	1.0658 (8)	0.4430 (6)	0.6682 (4)	0.0336 (18)	
H25	1.1293	0.3945	0.6394	0.040*	
C26	1.1067 (8)	0.5612 (6)	0.6726 (3)	0.0285 (16)	
H26	1.2012	0.5948	0.6473	0.034*	
C27	1.0130 (7)	0.6332 (5)	0.7133 (3)	0.0256 (16)	
H27	1.0454	0.7150	0.7159	0.031*	
C28	0.8440 (8)	0.8522 (5)	0.6049 (3)	0.0220 (15)	
C29	0.9322 (7)	0.8466 (5)	0.5402 (3)	0.0228 (15)	
C30	1.1031 (8)	0.8200 (5)	0.5399 (4)	0.0330 (17)	
H30	1.1607	0.8058	0.5824	0.040*	
C31	1.1880 (8)	0.8145 (6)	0.4785 (4)	0.0381 (18)	
H31	1.3041	0.7947	0.4783	0.046*	
C32	1.1051 (9)	0.8377 (5)	0.4170 (4)	0.0345 (18)	
H32	1.1648	0.8348	0.3743	0.041*	
C33	0.9364 (9)	0.8650 (6)	0.4167 (4)	0.0363 (18)	
H33	0.8802	0.8816	0.3740	0.044*	
C34	0.8494 (8)	0.8681 (6)	0.4786 (3)	0.0314 (17)	
H34	0.7325	0.8850	0.4787	0.038*	
C35	0.6349 (12)	0.5821 (7)	0.5823 (4)	0.066 (3)	
H35A	0.5575	0.5189	0.6037	0.079*	
H35B	0.7123	0.6024	0.6182	0.079*	
C36	0.7341 (11)	0.5337 (7)	0.5223 (5)	0.077 (3)	
H36A	0.7981	0.4604	0.5399	0.115*	
H36B	0.8110	0.5965	0.5016	0.115*	
H36C	0.6566	0.5133	0.4871	0.115*	
Cl1	0.40407 (18)	0.80198 (13)	0.69429 (8)	0.0259 (4)	
N1	0.5147 (6)	0.9129 (4)	0.8316 (3)	0.0187 (12)	
N2	0.5372 (6)	1.0235 (4)	0.8556 (3)	0.0216 (12)	
N3	0.8537 (6)	0.9662 (4)	0.7779 (2)	0.0201 (12)	
N4	0.8238 (6)	1.0713 (4)	0.8082 (3)	0.0218 (12)	
N5	0.5673 (5)	1.0477 (4)	0.7025 (3)	0.0185 (12)	
N6	0.5746 (6)	1.1432 (4)	0.7404 (3)	0.0213 (12)	
N7	0.7767 (6)	0.8556 (4)	0.6573 (3)	0.0190 (12)	
O2	0.5442 (7)	0.6869 (5)	0.5561 (3)	0.0617 (16)	
H2'	0.4895	0.7142	0.5886	0.092*	
P1	0.74469 (19)	0.68826 (14)	0.80077 (9)	0.0189 (4)	
Ru1	0.65206 (6)	0.87432 (4)	0.74538 (3)	0.01676 (16)	

### Atomic displacement parameters (Å^2^)

**Table:**

	*U*^11^	*U*^22^	*U*^33^	*U*^12^	*U*^13^	*U*^23^
B1	0.027 (4)	0.015 (4)	0.025 (5)	0.004 (3)	−0.004 (4)	−0.008 (3)
C1	0.022 (3)	0.027 (4)	0.026 (4)	−0.002 (3)	0.000 (3)	−0.002 (3)
C2	0.034 (4)	0.039 (4)	0.019 (4)	0.002 (3)	0.007 (3)	0.000 (3)
C3	0.031 (4)	0.040 (4)	0.018 (4)	0.014 (3)	0.003 (3)	−0.014 (3)
C4	0.014 (3)	0.025 (4)	0.031 (4)	0.004 (3)	−0.001 (3)	0.002 (3)
C5	0.021 (3)	0.034 (4)	0.034 (5)	−0.004 (3)	−0.010 (3)	−0.001 (3)
C6	0.031 (4)	0.031 (4)	0.029 (4)	−0.012 (3)	−0.010 (3)	−0.004 (3)
C7	0.024 (3)	0.023 (4)	0.019 (4)	0.001 (3)	−0.007 (3)	−0.004 (3)
C8	0.031 (4)	0.032 (4)	0.026 (4)	0.006 (3)	−0.006 (3)	0.007 (3)
C9	0.022 (3)	0.012 (3)	0.034 (4)	0.000 (3)	0.001 (3)	0.002 (3)
C10	0.019 (3)	0.019 (3)	0.019 (4)	−0.006 (3)	−0.001 (3)	0.003 (3)
C11	0.024 (3)	0.029 (4)	0.022 (4)	−0.001 (3)	−0.003 (3)	0.002 (3)
C12	0.032 (4)	0.040 (4)	0.020 (4)	−0.003 (3)	0.000 (3)	−0.010 (3)
C13	0.032 (4)	0.050 (5)	0.019 (4)	−0.002 (3)	−0.009 (3)	0.002 (4)
C14	0.026 (4)	0.036 (4)	0.041 (5)	0.011 (3)	−0.012 (4)	0.000 (4)
C15	0.029 (4)	0.033 (4)	0.027 (4)	0.006 (3)	−0.007 (3)	−0.003 (3)
C16	0.023 (3)	0.016 (3)	0.019 (4)	0.004 (3)	0.006 (3)	0.003 (3)
C17	0.033 (4)	0.028 (4)	0.027 (4)	−0.007 (3)	−0.004 (3)	0.002 (3)
C18	0.040 (4)	0.033 (4)	0.025 (4)	−0.006 (3)	0.003 (4)	0.004 (3)
C19	0.032 (4)	0.024 (4)	0.036 (5)	−0.005 (3)	0.011 (4)	0.005 (3)
C20	0.021 (3)	0.023 (4)	0.040 (5)	−0.003 (3)	−0.001 (3)	−0.004 (3)
C21	0.028 (3)	0.023 (4)	0.023 (4)	0.005 (3)	−0.005 (3)	−0.001 (3)
C22	0.022 (3)	0.015 (3)	0.021 (4)	0.003 (3)	−0.003 (3)	−0.005 (3)
C23	0.034 (4)	0.041 (4)	0.026 (4)	0.000 (3)	0.005 (3)	−0.009 (4)
C24	0.040 (4)	0.028 (4)	0.043 (5)	−0.004 (3)	0.005 (4)	−0.007 (4)
C25	0.044 (4)	0.030 (4)	0.028 (5)	0.008 (3)	0.002 (4)	−0.012 (3)
C26	0.026 (3)	0.031 (4)	0.028 (4)	−0.002 (3)	0.012 (3)	−0.002 (3)
C27	0.023 (3)	0.023 (4)	0.032 (4)	−0.002 (3)	0.000 (3)	−0.006 (3)
C28	0.025 (3)	0.024 (4)	0.018 (4)	−0.002 (3)	−0.005 (3)	−0.004 (3)
C29	0.026 (3)	0.019 (3)	0.023 (4)	−0.002 (3)	0.003 (3)	0.001 (3)
C30	0.037 (4)	0.029 (4)	0.032 (5)	0.004 (3)	0.003 (4)	0.001 (3)
C31	0.029 (4)	0.045 (5)	0.039 (5)	0.009 (3)	0.007 (4)	−0.002 (4)
C32	0.046 (4)	0.033 (4)	0.026 (5)	−0.013 (3)	0.011 (4)	−0.011 (3)
C33	0.046 (4)	0.047 (5)	0.018 (4)	−0.020 (4)	−0.004 (4)	−0.005 (4)
C34	0.024 (3)	0.046 (4)	0.023 (4)	−0.005 (3)	−0.002 (3)	0.001 (4)
C35	0.105 (8)	0.028 (5)	0.065 (7)	−0.005 (5)	−0.042 (6)	0.002 (5)
C36	0.087 (7)	0.049 (6)	0.100 (9)	0.022 (5)	−0.035 (7)	−0.028 (6)
Cl1	0.0209 (8)	0.0269 (9)	0.0301 (11)	−0.0032 (7)	−0.0085 (8)	−0.0020 (8)
N1	0.016 (3)	0.019 (3)	0.020 (3)	0.002 (2)	−0.006 (2)	0.002 (2)
N2	0.024 (3)	0.024 (3)	0.018 (3)	0.001 (2)	−0.001 (3)	−0.005 (2)
N3	0.022 (3)	0.022 (3)	0.016 (3)	−0.001 (2)	−0.001 (2)	0.001 (2)
N4	0.023 (3)	0.019 (3)	0.024 (3)	0.000 (2)	−0.003 (3)	−0.009 (2)
N5	0.016 (3)	0.017 (3)	0.023 (3)	0.002 (2)	−0.004 (2)	−0.005 (2)
N6	0.027 (3)	0.016 (3)	0.020 (3)	0.000 (2)	0.000 (3)	−0.002 (2)
N7	0.019 (3)	0.017 (3)	0.020 (3)	−0.002 (2)	−0.006 (3)	0.001 (3)
O2	0.066 (4)	0.073 (4)	0.046 (4)	0.007 (3)	−0.011 (3)	−0.009 (3)
P1	0.0193 (8)	0.0204 (9)	0.0169 (10)	0.0008 (7)	−0.0017 (8)	−0.0011 (8)
Ru1	0.0162 (2)	0.0176 (3)	0.0165 (3)	0.00072 (19)	−0.0021 (2)	−0.0017 (2)

### Geometric parameters (Å, °)

**Table:**

B1—N2	1.530 (9)	C20—C21	1.377 (8)
B1—N4	1.541 (8)	C20—H20	0.9500
B1—N6	1.545 (8)	C21—H21	0.9500
B1—H1'	1.0000	C22—C27	1.383 (8)
C1—N1	1.326 (8)	C22—C23	1.397 (8)
C1—C2	1.380 (8)	C22—P1	1.838 (6)
C1—H1	0.9500	C23—C24	1.384 (8)
C2—C3	1.365 (9)	C23—H23	0.9500
C2—H2	0.9500	C24—C25	1.382 (9)
C3—N2	1.341 (7)	C24—H24	0.9500
C3—H3	0.9500	C25—C26	1.361 (8)
C4—N3	1.332 (7)	C25—H25	0.9500
C4—C5	1.378 (8)	C26—C27	1.382 (8)
C4—H4	0.9500	C26—H26	0.9500
C5—C6	1.379 (8)	C27—H27	0.9500
C5—H5	0.9500	C28—N7	1.144 (7)
C6—N4	1.339 (7)	C28—C29	1.436 (9)
C6—H6	0.9500	C29—C34	1.375 (8)
C7—N5	1.323 (7)	C29—C30	1.393 (8)
C7—C8	1.385 (8)	C30—C31	1.366 (9)
C7—H7	0.9500	C30—H30	0.9500
C8—C9	1.356 (8)	C31—C32	1.375 (8)
C8—H8	0.9500	C31—H31	0.9500
C9—N6	1.355 (7)	C32—C33	1.377 (9)
C9—H9	0.9500	C32—H32	0.9500
C10—C15	1.386 (7)	C33—C34	1.380 (9)
C10—C11	1.396 (8)	C33—H33	0.9500
C10—P1	1.833 (6)	C34—H34	0.9500
C11—C12	1.372 (8)	C35—O2	1.403 (8)
C11—H11	0.9500	C35—C36	1.535 (11)
C12—C13	1.380 (8)	C35—H35A	0.9900
C12—H12	0.9500	C35—H35B	0.9900
C13—C14	1.364 (8)	C36—H36A	0.9800
C13—H13	0.9500	C36—H36B	0.9800
C14—C15	1.383 (8)	C36—H36C	0.9800
C14—H14	0.9500	Cl1—Ru1	2.4259 (14)
C15—H15	0.9500	N1—N2	1.371 (6)
C16—C17	1.383 (8)	N1—Ru1	2.063 (5)
C16—C21	1.400 (7)	N3—N4	1.373 (6)
C16—P1	1.834 (6)	N3—Ru1	2.069 (4)
C17—C18	1.381 (8)	N5—N6	1.354 (6)
C17—H17	0.9500	N5—Ru1	2.102 (5)
C18—C19	1.362 (8)	N7—Ru1	1.991 (5)
C18—H18	0.9500	O2—H2'	0.8400
C19—C20	1.383 (8)	P1—Ru1	2.3205 (16)
C19—H19	0.9500		
			
N2—B1—N4	108.7 (5)	C24—C25—H25	120.7
N2—B1—N6	107.5 (5)	C25—C26—C27	121.1 (6)
N4—B1—N6	107.0 (5)	C25—C26—H26	119.5
N2—B1—H1'	111.1	C27—C26—H26	119.5
N4—B1—H1'	111.1	C26—C27—C22	121.5 (6)
N6—B1—H1'	111.1	C26—C27—H27	119.3
N1—C1—C2	111.4 (6)	C22—C27—H27	119.3
N1—C1—H1	124.3	N7—C28—C29	178.6 (7)
C2—C1—H1	124.3	C34—C29—C30	119.9 (6)
C3—C2—C1	104.6 (6)	C34—C29—C28	120.5 (5)
C3—C2—H2	127.7	C30—C29—C28	119.6 (6)
C1—C2—H2	127.7	C31—C30—C29	120.0 (7)
N2—C3—C2	109.1 (6)	C31—C30—H30	120.0
N2—C3—H3	125.5	C29—C30—H30	120.0
C2—C3—H3	125.5	C30—C31—C32	119.9 (6)
N3—C4—C5	110.9 (5)	C30—C31—H31	120.1
N3—C4—H4	124.5	C32—C31—H31	120.1
C5—C4—H4	124.5	C31—C32—C33	120.6 (7)
C6—C5—C4	104.9 (5)	C31—C32—H32	119.7
C6—C5—H5	127.6	C33—C32—H32	119.7
C4—C5—H5	127.6	C32—C33—C34	119.8 (6)
N4—C6—C5	108.8 (5)	C32—C33—H33	120.1
N4—C6—H6	125.6	C34—C33—H33	120.1
C5—C6—H6	125.6	C29—C34—C33	119.8 (6)
N5—C7—C8	110.7 (5)	C29—C34—H34	120.1
N5—C7—H7	124.7	C33—C34—H34	120.1
C8—C7—H7	124.7	O2—C35—C36	108.7 (7)
C9—C8—C7	105.0 (6)	O2—C35—H35A	110.0
C9—C8—H8	127.5	C36—C35—H35A	110.0
C7—C8—H8	127.5	O2—C35—H35B	110.0
N6—C9—C8	108.6 (5)	C36—C35—H35B	110.0
N6—C9—H9	125.7	H35A—C35—H35B	108.3
C8—C9—H9	125.7	C35—C36—H36A	109.5
C15—C10—C11	117.4 (5)	C35—C36—H36B	109.5
C15—C10—P1	122.4 (5)	H36A—C36—H36B	109.5
C11—C10—P1	120.1 (4)	C35—C36—H36C	109.5
C12—C11—C10	121.4 (5)	H36A—C36—H36C	109.5
C12—C11—H11	119.3	H36B—C36—H36C	109.5
C10—C11—H11	119.3	C1—N1—N2	105.8 (5)
C11—C12—C13	120.4 (6)	C1—N1—Ru1	136.1 (4)
C11—C12—H12	119.8	N2—N1—Ru1	118.0 (4)
C13—C12—H12	119.8	C3—N2—N1	109.1 (5)
C14—C13—C12	118.8 (6)	C3—N2—B1	129.9 (5)
C14—C13—H13	120.6	N1—N2—B1	120.8 (5)
C12—C13—H13	120.6	C4—N3—N4	106.3 (5)
C13—C14—C15	121.4 (6)	C4—N3—Ru1	134.6 (4)
C13—C14—H14	119.3	N4—N3—Ru1	118.6 (3)
C15—C14—H14	119.3	C6—N4—N3	109.2 (5)
C14—C15—C10	120.6 (6)	C6—N4—B1	129.9 (5)
C14—C15—H15	119.7	N3—N4—B1	120.0 (4)
C10—C15—H15	119.7	C7—N5—N6	106.7 (4)
C17—C16—C21	117.1 (6)	C7—N5—Ru1	133.9 (4)
C17—C16—P1	123.2 (5)	N6—N5—Ru1	119.3 (4)
C21—C16—P1	119.5 (5)	N5—N6—C9	109.0 (5)
C18—C17—C16	121.8 (6)	N5—N6—B1	119.1 (4)
C18—C17—H17	119.1	C9—N6—B1	132.0 (5)
C16—C17—H17	119.1	C28—N7—Ru1	175.5 (5)
C19—C18—C17	120.7 (7)	C35—O2—H2'	109.5
C19—C18—H18	119.7	C10—P1—C16	100.4 (3)
C17—C18—H18	119.7	C10—P1—C22	102.2 (3)
C18—C19—C20	118.8 (6)	C16—P1—C22	99.5 (3)
C18—C19—H19	120.6	C10—P1—Ru1	113.87 (19)
C20—C19—H19	120.6	C16—P1—Ru1	119.88 (18)
C21—C20—C19	121.0 (6)	C22—P1—Ru1	118.0 (2)
C21—C20—H20	119.5	N7—Ru1—N1	173.57 (19)
C19—C20—H20	119.5	N7—Ru1—N3	89.01 (19)
C20—C21—C16	120.7 (6)	N1—Ru1—N3	90.33 (18)
C20—C21—H21	119.7	N7—Ru1—N5	89.35 (19)
C16—C21—H21	119.7	N1—Ru1—N5	84.21 (19)
C27—C22—C23	117.2 (6)	N3—Ru1—N5	85.23 (17)
C27—C22—P1	120.7 (4)	N7—Ru1—P1	94.34 (14)
C23—C22—P1	122.1 (5)	N1—Ru1—P1	92.08 (13)
C24—C23—C22	120.7 (7)	N3—Ru1—P1	92.67 (13)
C24—C23—H23	119.7	N5—Ru1—P1	175.72 (15)
C22—C23—H23	119.7	N7—Ru1—Cl1	88.69 (13)
C23—C24—C25	120.9 (6)	N1—Ru1—Cl1	90.79 (13)
C23—C24—H24	119.6	N3—Ru1—Cl1	169.39 (13)
C25—C24—H24	119.6	N5—Ru1—Cl1	84.39 (13)
C26—C25—C24	118.6 (6)	P1—Ru1—Cl1	97.83 (5)
C26—C25—H25	120.7		
