# (Benzonitrile-κ*N*)chlorido[hydrido­tris(pyrazol-1-yl-κ*N* ^2^)borato](triphenyl­phosphine-κ*P*)ruthenium(II) ethanol solvate. Corrigendum

**DOI:** 10.1107/S1600536809016183

**Published:** 2009-05-07

**Authors:** Hung-Chun Tong, Po-Yo Wang, Yu-Lin Kuo, Chia-Her Lin, Yih-Hsing Lo

**Affiliations:** a Department of Chemical Engineering, Tatung University, Taipei 104, Taiwan; b Department of Natural Science, Taipei Municipal University of Education, Taipei 10048, Taiwan; c Department of Materials Engineering, Tatung University, Taipei 106, Taiwan; d Department of Chemistry, Chung Yuan Christian University, Chung Li 32023, Taiwan

## Abstract

The author list in the paper by Tong, Hung, Wang, Lin & Lo [*Acta Cryst.* (2009), E**65**, m438] is corrected.

## Abstract 2

Corrigendum to *Acta Cryst.* (2009), E**65**, m438.

In the paper by Tong, Hung, Wang, Lin & Lo [*Acta Cryst.* (2009), E**65**, m438], the author list was incorrect. The correct author list is given above.

